# Liposomal Cytarabine as Cancer Therapy: From Chemistry to Medicine

**DOI:** 10.3390/biom9120773

**Published:** 2019-11-23

**Authors:** Bahare Salehi, Zeliha Selamoglu, Ksenija S. Mileski, Raffaele Pezzani, Marco Redaelli, William C. Cho, Farzad Kobarfard, Sadegh Rajabi, Miquel Martorell, Pradeep Kumar, Natália Martins, Tuhin Subhra Santra, Javad Sharifi-Rad

**Affiliations:** 1 Student Research Committee, School of Medicine, Bam University of Medical Sciences, Bam 44340847, Iran; bahar.salehi007@gmail.com; 2 Department of Medical Biology, Faculty of Medicine, Nigde Ömer Halisdemir University, Campus, 51240 Nigde, Turkey; zselamoglu@ohu.edu.tr; 3 Department of Morphology and Systematic of Plants, Institute of Botany and Botanical Garden “Jevremovac,” Faculty of Biology, University of Belgrade, Belgrade 11000, Serbia; ksenija.mileski@bio.bg.ac.rs; 4 O.U. Endocrinology, Department of Medicine (DIMED), University of Padova, via Ospedale 105, 35128 Padova, Italy; 5 AIROB, Associazione Italiana per la Ricerca Oncologica di Base, 35128 Padova, Italy; marcoredaelli@email.it; 6 Venetian Institute for Molecular Science and Experimental Technologies, VIMSET. Pz Milani, 4 30010 Liettoli di Campolongo Maggiore, VE, Italy; 7 Department of Clinical Oncology, Queen Elizabeth Hospital, Hong Kong SAR, China; 8 Phytochemistry Research Center, Shahid Beheshti University of Medical Sciences, Tehran 1991953381, Iran; farzadkf@yahoo.com; 9 Department of Medicinal Chemistry, School of Pharmacy, Shahid Beheshti University of Medical Sciences, Tehran 11369, Iran; 10 Department of Clinical Biochemistry, School of Medicine, Shahid Beheshti University of Medical Sciences, Tehran 23871, Iran; sadegh.rajabi2017@gmail.com; 11 Department of Nutrition and Dietetics, Faculty of Pharmacy, University of Concepcion, Concepcion 4070386, Chile; mmartorell@udec.cl; 12 Universidad de Concepción, Unidad de Desarrollo Tecnológico, UDT, Concepcion 4070386, Chile; 13 Department of Forestry, North Eastern Regional Institute of Science and Technology, (Deemed To Be University-MHRD), Nirjuli (Itanagar) 791109, India; pkbiotech@gmail.com; 14 Faculty of Medicine, University of Porto, Alameda Prof. Hernâni Monteiro, 4200-319 Porto, Portugal; ncmartins@med.up.pt; 15 Institute for Research and Innovation in Health (i3S), University of Porto, 4200-135 Porto, Portugal; 16 Department of Engineering Design, Indian Institute of Technology Madras, Chennai 600036, India; tuhin@iitm.ac.in; 17 Zabol Medicinal Plants Research Center, Zabol University of Medical Sciences, Zabol 61615-585, Iran

**Keywords:** DepoCyt, liposomal cytarabine, liposomal ara-C, liposomal cytosine arabinoside, chemotherapy, cancer therapy

## Abstract

Cancer is the second leading cause of death worldwide. The main modality to fight against cancer is surgery, radiotherapy, and chemotherapy, and more recently targeted therapy, gene therapy and immunotherapy, which play important roles in treating cancer patients. In the last decades, chemotherapy has been well developed. Nonetheless, administration of the drug is not always successful, as limited drug dosage can reach the tumor cells.. In this context, the possibility to use an encapsulated anti-cancer drug may potentially solve the problem. Liposomal cytarabine is a formulation with pronounced effectiveness in lymphomatous meningitis and reduced cardiotoxicity if compared to liposomal anthracyclines. Thus, the future liposomal cytarabine use could be extended to other diseases given its reduction in cytotoxic side effects compared to the free formulation. This review summarizes the chemistry and biology of liposomal cytarabine, with exploration of its clinical implications.

## 1. Introduction

Cancer has a major impact on society across the world. In 2018, an estimated 1.7 million new cases of cancer will be diagnosed in the United States, and about six hundred thousand people will die from the disease [1]. The major cause of cancer-related mortality is metastasis, for which a curative approach is still lacking. Moreover, the use of chemotherapy is useful also in systemic treatment; regardless of cancer has been metastasized [2]. However, drug delivery may not effectively target the desired site or may have effects on healthy tissues causing adverse effects [3,4,5]. Thus, various formulations for chemotherapeutic drugs have been developed [6,7].

Liposomes had been first described by a British hematologist Dr. Alec D. Bangham in 1961 at the Babraham Institute, Cambridge. These were discovered when Dr. Alec Bangham and R. W. Horne added a negative stain to dry phospholipids when testing the institute’s new electron microscope. Two Greek words, ‘lipo’ (fat) and ‘soma’ (body), compose the word liposome and it is named so because of its composition is similar to phospholipids. By encapsulating in spherical multivesicular, biodegradable lipid-based particles known as DepoCyt, liposomal cytarabine is released gradually after administration, thereby prolonging exposure of this drug in cerebrospinal fluid.

When querying Scopus database for “liposomal cytarab”, more than 550 documents were retrieved, as the terms appeared first in 1990. From 2007 to 2013, there has been an exponential increase of publications, while most of the documents were from the United States, followed by Germany and Italy. Almost half of the documents belong to the field of medicine, and nearly a quarter from biochemistry and pharmacology. Moreover, we searched for liposomal cytarabine in different databases, such us PubMed, Scopus, Web of Science, Embase, Google Scholar. For this review we used these keywords: liposomal Ara-C, liposomal cytosine arabinoside, liposomal cytarabine, DepoCyt, DepoCyte.

It is well reported that neoplastic meningitis is a feared complication in cancer patients, the median survival ranging from some weeks to a few months. Management lie on palliative and aims to provide symptoms of relief while delaying neurological deterioration. Intrathecal methotrexate and liposomal cytarabine is the most widely used treatment in such clinical situations.

This article aims at discussing liposomal cytarabine in cancer therapy, starting from chemistry, dealing with its source and bioavailability, and/or coming to preclinical studies as well as clinical trials. A summarizing table can be consulted for an immediate overview (Table 1).

## 2. Chemistry of Liposomes: From Models to New Applications

DepoCyt has been developed as a liposomal drug to better understand its therapeutic role in humans and to explore the mechanism of DepoCyt configuration.

The setup of artificial models for the study of liposome has been developed with the primary aim of an in vitro reconstitution of natural membrane functions. This was important for a deep investigation of chemoreception and even in sensor engineering. Over the years, scientific literature of artificial lipid membranes and, of course, liposomes, expanded and went beyond the initial purposes [8]. In this paragraph, we focused on the state-of-the-art of artificial lipid models by discussing their multiple applications and future perspectives [9,10].

One of the most intriguing fields of application for artificial membranes is drug delivery. The first demonstration of its potential was with catecholamines in buffer-induced pH gradient from the inner part of the liposome to the external environment [11]. This buffering method was then successfully applied in the study of doxorubicin, idarubicin, and daunorubicin; up to now, differences in liposome compound release were reported in relation with the hydrophobicity of the tested drugs [12,13]. Different approaches were reported to promote pH gradients using ionophores such as calcium acetate and ammonium sulfate [14]. Moreover, when the tested drug was not compatible with the buffer/pH approach (for example, hydrophobic molecules) the transition through metal complexation was proven to be a good alternative model [15].

In addition to the pH gradients, functionalization of liposome surface may be required to improve drug targeting. The most common approach involves the insertion of lipid-ligand conjugates within the liposome membrane, either before (in situ) or following liposome formation [16]. When this approach is not possible or suitable, the use of compounds inducing the functionalization of the lipid membrane has been demonstrated to be effective [17].

These methods are still experimental and non-specific ligand-drug interactions are yet to be addressed. In the modern approach ligands are designed to target membrane molecules mainly expressed in cancer cells, this results in an increased affinity and in enhanced tailored internalization [15]. As a further advancement in liposome technology in cell–cell interaction experiments, colloidal core–shell for lipid bilayer membranes were used for their stability and in a wide range of experimental conditions [18] due to the structural electrostatic interactions [19]. Also, the usage of nano-cores allowed the monitoring of liposome-core interactions at the molecular level even in cryoelectron microscopy [20]. Nevertheless, this approach opened to the study of self-assembly dynamics revealing potentially critical steps in the model [17,18].

Moving into the direction of drug discovery, the development of tools compatible with the physiology of the host is the most intriguing topic. In the design of therapeutic liposomal approaches to intracellular target, the interaction between the liposome and the lipid membrane can play a relevant role in drug pharmacokinetics and in the consequent effectiveness of the treatment [21]. For example, intracellular accumulation was reported in an experiment with quinolones such as ciprofloxacin [22]. Then, these molecules demonstrated to be strongly interactive with liposome membrane leading to condensation, and even with minimal differences in molecular structure when involved in lipophilicity alteration. [23].

Other compounds, such as azithromycin, are involved in chemical interaction with a specific category of lipids. This was demonstrated in morphometric experiments in which the effect on the bilayer was recorded only with phospholipid, but not with sphingomyelin [24]. Further experiments explained the mechanism of interaction between azithromycin and the bilayer that specifically decreased the lipid cohesion affecting the membrane elasticity [24]. This revealed a new possible application in liposome-drug delivery using chemicals to induce drug incorporation.

The determination of drug efficiency in a membrane–drug interaction system could be affected by various biases especially in the early phases and with charged molecules [25]. To overcome these problems, different models based on ionic interaction on liposome membranes were set up [21]. Liposome models revealed to be particularly suitable in the analysis of passive drug delivery through membranes [26]. For example, amphiphiles drugs were demonstrated to form micelles via hydrolyzation of ester links in phospholipid of membranes via a catalytic reaction. This reaction can alter the phospholipid membrane and makes it fragment these results in micelle formation; this mechanism is the first step of the drug penetration process [27].

Currently, many membrane delivery systems are based on drug-coated polymers, most of them under patent. This technology is specifically designed for the interaction with phospholipidic membranes and of course tested on artificial liposomes. A common mechanism of delivery is the electrostatic interaction with lipid head which results in modulation of drug entrance into the cytoplasm [28]. The use of these new approaches with reactive molecular species in cancer treatment is still an ongoing issue and is currently investigated in membrane models even at the atomic level. The theory followed in this case is different: the main goal is the formation of pores for the delivery of the reactive molecules into cells by electric interaction [29].

Nanocolloids are emerging technologies with the capabilities to target intracellular areas. These substances are naturally unstable and very difficult to manage in dynamic biological systems: this is due to the elevated energy level and the tendency to particle aggregation [30]. Modification of nanocolloids surface can overcome many of these problems, but this approach could not be considered as definitive due to a variety of the possible outcomes (as unpredictable aggregation) [31]. In particular, unpredictable aggregation could be solved using water-soluble polymers coat [32] or lipid encapsulation [33], although these approaches cannot overcome the intrinsic instability [34,35]. The routinely use of these innovations remains a challenge: however, the development of new strategies can address a better knowledge of artificial membrane applications. As collateral evolution of these systems, the use of encapsulated copolymer micelles conjugated to DNA was reported in the embryogenetic study as a new promising tool [36].

The study and analysis of drug permeability is a hinge point in the drug development process and is one of the most debated issues for future application of liposome technology. Preclinical and clinical results on nanoformulated anti-cancer substances revealed promising results. The lower toxicity and great efficacy of these systems compared to conventional chemotherapy will be strongly appreciated [37]. However, one of the most challenging problems the drug development process faces is bioavailability.

## 3. Bioavailability and Sources

**Figure 1:**
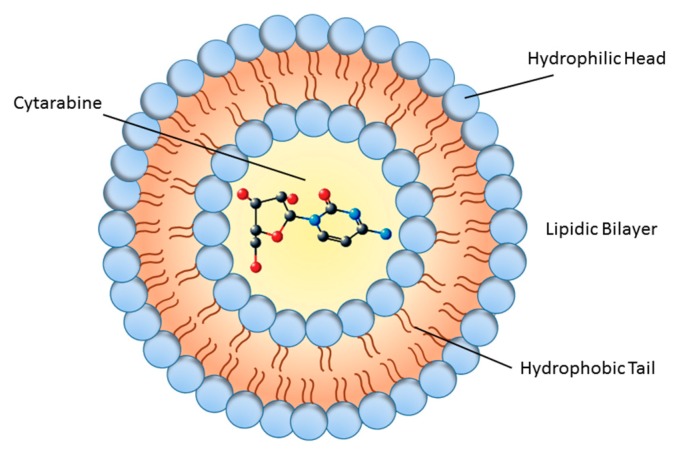
Schematic diagram of liposome structure. The inner part shows the cytarabine molecule.

Generally, there are multiple challenges in formulating therapeutic molecules for sustained delivery to target organs. These bottlenecks include: (1) protecting the structural integrity of the therapeutic molecule to maintain its bioactivity and stability, (2) obtaining high concentrations of drug in the delivery particle to reach high bioavailability, (3) maintaining sustained blood drug concentrations within the therapeutic range for an extended period of time without a burst effect (the risk of exposing the patient to a local or temporal drug overdose [38], (4) checking drug release length to meet a dosage regimen to accommodate with therapeutic needs, and (5) producing in vivo biological effects that can be sustained during the desired period of time. Liposomal cytarabine can be considered a carrier system that meets these challenges providing the sustained delivery of molecules to target sites [39,40]. Accordingly, augmented permeability and retention, alleviated toxicity, and improved pharmaceutical characteristics—such as bioavailability, stability, and solubility—are major advantages of nano drugs [41]. DepoCyt is composed of lipid nanoparticles that contain Ara-C (4-amino-1–β -d-arabinofuranosyl-2(1*H*)-pyrimidinone, also known as cytosine arabinoside), which is a cytosine analog with arabinose sugar that kills cancer cells by interfering with DNA synthesis [42]. DepoCyt is administered via direct injection into the spinal canal, or into the subarachnoid space for the intrathecal treatment of lymphomatous meningitis [43]. Ara-C has a short plasma half-life, low lipophilicity and stability, and limited bioavailability. Some efforts have been devoted to enhancing the low bioavailability and stability of Ara-C [44]. These efforts can be divided into two major categories depending upon formulation and modification: prodrugs and drug delivery systems. The prodrug approach for Ara-C includes chemical modification or incorporation of a potentiated group on parent drug, while in the delivery system approach, the medication is physically encapsulated without any chemical modification [45]. The DepoCyt formulation consists of a sterile suspension of Ara-C which is encapsulated into multivesicular lipid-based polymeric liposomal carriers composed of cholesterol, glycerol trioleate, triglyceride, phospholipids (dipalmitoyl phosphatidylglycerol), and dioleoyl phosphatidylcholine (Figure 1) [46].

It is well known that liposomal particles, among the effective delivery systems, are successful in increasing the Ara-C half-life and consequently in the treatment of lymphomatous meningitis (see Section 6) [45]. Encapsulated Ara-C provides continuous exposure to cytotoxic concentrations of this drug (>0.02 L g/mL) to tumor cells. Sustained release of Ara-C from these carriers provides a prolonged drug exposure, leading to lower Ara-C peak concentrations and to its protracted release in comparison to standard Ara-C [47]. DepoCyt has been shown to extend the half-life of Ara-C in the central nervous system (CNS), leading to improved patient response and reduced disease progression [48].

## 4. Cytarabine Nanoparticles in Preclinical Settings

Nanoparticles are considered specific chemical formulations with a diameter comprised approximately between 1 and 100 nm [49]. Among this plentiful group resides liposomes, which are the main form of nanoparticles used for the preparation of cytarabine in human clinical trials and are thoroughly explored in this review. Nonetheless, few works investigated the use of cytarabine nanoparticles different from liposomes in preclinical studies.

For example, cytarabine was conjugated in a self-assembling nanoparticle which included glutathione cleavable disulfide bond [44]. This combination resulted in a redox-sensitive drug–drug conjugate, more cell membrane permeable and more resistant to biological inactivation before arrival into tumor tissue. The effects were studied in B16F10 (mouse skin melanoma) and HT-29 (human colorectal cancer) cell lines, with increased cytotoxicity and apoptotic rate. Moreover, in B16F10 xenografted mice, the effects of cytarabine nanoparticles reduced tumor size and weight. Magnetic nanoparticles made by Fe_3_O_4_@SiO_2_ were obtained via chemical coprecipitation reaction and coating silica and produced a novel nanoparticle that was subsequently covered by cytarabine [50]. Cytotoxicity was evaluated in HL60 (human leukemia) cell line and showed a 2 times better efficacy if compared to cytarabine alone. Furthermore, cytarabine was conjugated to a glucose-functionalized amphiphilic random terpolymer, showing an internalization of this nanocarrier in HepG2 (human hepatoma) cell line [51]. The effects on cell viability confirmed the high ability of inhibiting tumor cell growth. On the same line, a novel nanoformulation of cytarabine prepared by polysorbate 80 lipid conjugate was able to reduce cell viability of EL-4 cells, a model of murine T cell lymphoma [52]. Another work analyzed the encapsulation efficiency of cytarabine (and methotrexate) obtained by modified reverse-phase evaporation method through the use of the UV–vis and NMR [53]. The authors showed that cytarabine encapsulation efficiency was 86.30% and suggested that this was a novel stable and potentially therapeutic preparation of cytarabine liposomes. Differently, a self-assembly multidrug copolymer loaded with cytarabine was prepared from random copolymer and the loading efficiency was 28.7 wt % [54]. In another research gelatin type, A nanoparticles crosslinked with genipin were loaded with cytarabine [55]. The UV spectral study demonstrated the stability and integrity of cytarabine even in highly acidic medium. Similarly PEGylated poly(lactic-co-glycolic acid) nanoparticles were loaded with cytarabine, again underling the ability of this new nanocarrier to be stable and with sustained phase release [56]; in addition, this nanoformulation was greatly internalized by the L1210 (mouse lymphocytic leukemia) cells suggesting beneficial effects in leukemia therapy [57].

## 5. Liposomal Drugs in Cancer

Cancer is one of the most destructive diseases and a leading cause of death in the world. Many therapeutic strategies are available to treat cancer such as chemotherapy, radiation treatment, surgery, etc. [58,59]. Combination therapy is a well-known and used approach for the treatment of cancer. The co-delivery of chemotherapeutic drugs and genes can provide a promising strategy to overcome drug resistance in cancer therapy.

Nanotechnology has an important function in cancer therapy indeed nanovectors such as liposomes, micelles, carbon nanotubes, metal nanoparticles, dendrimers, and natural and polymer-drug conjugates can be used. Nanotechnology is the application of a scientific knowledge that can transport drugs between different environments, and setting and liposomes are concrete examples of nanotechnology. They show great promise, among the various investigated delivery systems. Nanotechnology covers several research fields [60,61,62], but one area—nanoparticle-based drug delivery—has the potential to solve some limitations encountered in cancer therapies. The basic target of liposomal drug delivery is to deliver the therapeutic agent preferentially near to the tumor site through the improved permeability effect [63]. Ligand-targeted liposomes have the potential to impact the development of new therapies to cancer treatment. However, these highly engineered liposomes have been produced new problems, such as accelerated clearance from circulation, compromised targeting owing to non-specific serum protein binding, and hindered tumor penetration [63].

The first clinical interesting work on liposomes analyzed liposomal amphotericin B (Ambisome1), which was used for fungal infections. This drug system received clinical approval in 1990. Liposomes are frequently obtained from naturally occurring phospholipids and cholesterol. Liposomal nanoparticles have been designed to be multifunctional, with different components that provide control over such characters as permeability, biodistribution, elimination half-lives, and targeting specificity [64]. Moreover, liposomes have poor extravasation in tissues with tight endothelial junctions, and this fact can result in side effects decrease of the liposomal drug if compared to the free drug [65].

The first report of the improved in vivo activity of liposome-entrapped drugs conducted in animal models used cytosine arabinoside, an anti-cancer drug, which significantly increased survival time of mice with leukemia. Consequently, this became a popular model system for analyzing the effects of liposomes on therapeutic outcomes [65].

Numerous anticancer drugs have inappropriate pharmaceutical and pharmacological features such as low aqueous solubility, irritant properties, lack of stability, rapid metabolism, unfavorable pharmacokinetics, and non-selective drug distribution [64]. Moreover, these features can lead to adverse consequences such as lack of or suboptimal therapeutic activity, dose-limiting side effects and poor patient quality of life [64]. Nanoscale drug delivery systems, defined as drug delivery systems with particle diameters of approximately 100 nm or less, are a promising technology which is attracting considerable attention as a means of overcoming most of the limitations of conventional anticancer drugs. Generally, conventional drugs are small molecules under 500 Da, and these small molecules can be quickly affected by clearance and suboptimal distribution, resulting in toxic side effects. Also, high polarity drugs commonly have low intracellular absorption and limited effects. Therefore, drug encapsulation in delivery systems is an efficient approach to improve the pharmacokinetics of hydrophilic drugs. In the past years, hydrophilic drug encapsulation in polymer-based nanoparticles has shown that this technology could provide a better pharmacokinetic profile and bioavailability, increasing the therapeutic effect and reducing toxicity compared to standard drug [43]. It is worth noting that the reticuloendothelial system can clear liposomes as they can be bound by proteins, such as complement and immunoglobulins.

The liposomal co-delivery system has become a promising technology for cancer therapies. This system is especially challenging regarding costs, and it seems that pharmaceutical companies are interested in developing this new type of drug. The biggest challenge in drugs and gene agents co-delivering is to obtain applicable carriers, since gene agents have a higher molecular weight and negatively charged surface, while mostly used anti-cancer drugs are small hydrophobic molecules [59]. Liposome-based drug delivery systems have been used in several experimental cancer-research and clinical trials [66], and various patents are based on the same [67]. Some examples of the active ingredients of the liposomal formulations used as anti-cancer treatment are daunorubicin [68], doxorubicin [69,70], all-*trans* retinoic acid [71], mitoxantrone [72], irinotecan [73], and paclitaxel [74].

Parallel research has improved the stability and efficiency of drug entrapment in liposomes, particularly regarding cationic amphiphiles which have a long-circulating time and enhanced accumulation in tumors [75].

Nonetheless, limitations of active targeting of liposomes to tumor cells have been observed, from both formulation and/or pharmacology point of view. Liposomal systems can be clinically delusive since they are dynamic, constantly equilibrating, self-assembled entities whose shape and surface chemistry are ill-defined, especially when placed into the biological milieu where equilibration reactions occur with lipid membranes. [76].

More studies on liposome-encapsulated anticancer drugs are necessary to compare their increased efficacy and tolerability to their non-liposomal counter parts [64].

## 6. Liposomal Cytarabine

### 6.1. Preclinical Data and Research

The preclinical data continuously provide novel compounds and thus complement clinical studies with potentially valuable active drugs. In general, preclinical research is barely successfully translated into clinical practice: the difficulty also comes from the pathophysiologic differences in human cancers. Thus, drug delivery efficiency is limited by blood–tumor barrier permeability which depends on tumor type, size, and location.

In addition, the mechanism of action of liposomal cytarabine is strictly related to its main constituent, i.e., cytarabine that belongs to the class of antimetabolites. Cytarabine (molecular formula: C_9_H_13_N_3_O_5_) interferes with DNA synthesis, acting on DNA/RNA polymerase (and other nucleotide reductase enzymes), reducing cell ability to replicate [77]. Of course, with the addition of cytarabine to liposome, it is facilitated its entrance to the cell, as already described in paragraphs 2 and 3. Thus, the effects of cytarabine on cell cycle process play a key role on cell survival, blocking S phase.

This first work exploring the use of cytarabine dated back to 1961, when Evans and collaborators studied 1-β-d-Arabinofuranosylcytosine hydrochloride in mice tranplanted with Sarcoma 180, Ehrlich carcinoma, and L-1210 leukemia cells [78]. The authors showed a great mice response to the drug, even if the replication of experiments in rats led to no therapeutic effect, introducing an animal-sensibility. A couple of years later, 1-β-d-Arabinofuranosylcytosine hydrochloride was experimentally used in humans, where it induced a decrease of tumor masses in three patients affected by lymphosarcoma and where it was partially effective in 2 out of 10 treated patients with disseminated carcinomatosis [79].

**Figure 2:**
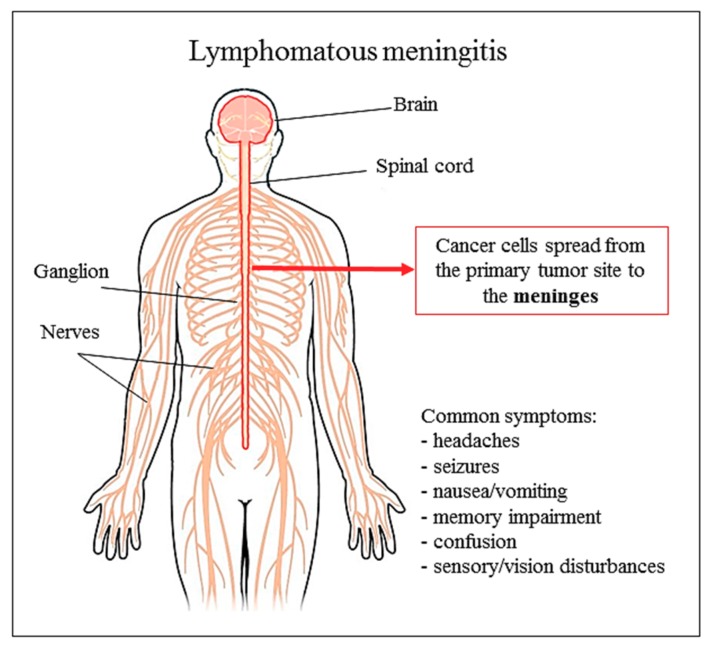
Lymphomatous meningitis and common symptoms.

Later, marine-derived natural product Ara-C was first used in human disease in 1974 [80,81]. Many liposomal nanotherapeutics are being evaluated preclinically, and it has been proved that they possess great potential in vitro and in vivo animal models. Liposomal carriers of many anti-neoplastic agents can increase anticancer efficacy, can protect drug degradation and can reduce its toxicity [82,83]. In such a way, a liposomal formulation of Ara-C (Figure 1) is approved and increasingly used as a very effective tool in the treatment of patients with leukemia or lymphomas [64]. Before liposomal Ara-C was introduced in the market as nanomedicine, DepoCyt was studied for clinical treatment of lymphomatous meningitis, starting from preclinical studies (Figure 2) [84]. As a part of preclinical development, liposomal Ara-C was tested in vivo in different animal models such as mice, rats, dogs, and primates [85,86]. Likewise, phase II/III studies for leukemia and phase I/II for glioblastoma have been completed. While the last study (NCT01044966) was terminated due to lack of adequate patient enrollment into trial, four studies were available for acute lymphoblastic leukemia. One was suspended (due to sterility problems in DepoCyt production), one was terminated (due to lack of adequate patient enrollment into trial), one was defined as ‘unknown’ (the principal investigator did not report necessary info or update the file), and only one (NCT00795756) had results which have been published in Haematologica [87]. This last study compared intrathecal DepoCyt with triple intrathecal therapy (TIT) (Methotrexate 12.5 mg + Cytarabine 50 mg + Prednisolone 40 mg injected intrathecally). The results showed that DepoCyt had higher neurotoxicity than TIT (CNS toxicity grade 3-4), but DepoCyt was still considered highly active against CNS leukemia, so that the authors suggested to use DepoCyt at reduced doses (15 or 25 mg rather than 50 mg), retaining significant pharmacological activity while having a safer toxicity profile. Moreover, intrathecal dexamethasone is fundamental to reduce side effects and should be administered to avoid overall neurotoxicity [88].

Previously, the sustained-release nature of the Ara-C liposomal preparations was confirmed in different studies [40,89,90,91]. Preclinical investigations of Kim et al. [90] in a nonhuman primate model (phase I study) demonstrated that the Ara-C sustained-release liposomal injection had a visible pharmacokinetic advantage compared with unencapsulated Ara-C, since the terminal half-life of free Ara-C was increased more than 40-fold (from 3.4 h to 141 h after a single intrathecal dose of DepoCyt). Still, the authors observed significant similarity in toxicities and side effects of intrathecal DepoCyt and free Ara-C such as fever, headache, back pain, nausea, and encephalopathy. In another study, Kohn et al. [91] investigated the distribution pattern, metabolism and excretion of radiolabeled Ara-C and the primary phospholipid component dioleoylphosphatidylcholine (^3^H-cytarabine, ^14^C-DOPC) of DepoCyt particles after lumbar intrathecal administration in spinally catheterized rat models. The authors quantified radioactivity in the central nervous system, peripheral tissues, cerebrospinal fluid, blood, urine, and feces at various time points up to 504 h. They concluded that after injection, both radiolabels distributed rapidly throughout the neuraxis and had similar biphasic kinetics profiles. Thus, levels of drug and lipid radiolabels declined in a biphasic manner from cerebrospinal fluid and plasma, with an initial rapid decline over the first 96 h, followed by a much slower rate of decline out to 504 h. The free Ara-C showed greater diffusion mobility in the central nervous system than the lipid particles according to the greater cisternal: lumbar ratio for ^3^H than for ^I4^C isotopes. ^3^H and ^14^C labels were subsequently distributed into the systemic circulation, and their concentrations had similar patterns in plasma and urine since both radioisotopes were detected 5 min after intrathecal dose administration. Their levels peaked at 160 min and then declined similarly, but they were still present at 504 h in both plasma and urine. The plasma analyses showed that the majority of radiolabeled lipids remained as phospholipids (>90%) and a smaller amount was metabolized to radioactive monoglycerides and fatty acids. In contrast, the ^3^H radiolabel occurred in plasma as metabolized Ara-C, uracil arabinoside. According to urine analyses results, more than 90% of the original ^3^H dose was excreted in urine, by Ara-C clearance in humans [92].

In contrast, the majority of the ^14^C-DOPC was catabolized, and the greatest percentage of the phospholipids was expired as ^14^CO_2_. Smaller doses remained incorporated in the central nervous system (7%) and peripheral tissues (8%), while only 6% of the ^14^C dose was excreted in the urine. Systematic examination of a variety of peripheral organs revealed low (approximately 10% of the dose) or background concentrations of both followed isotopes at all-time points. Finally, a very low level of ^3^H, but not ^14^C, was found in feces [90].

To determinate the optimal route to deliver ^14^C-cytosine arabinoside (Ara-C), Groothuis et al. [80] evaluated the intravenous, intrathecal, and intraventricular infusions and convection-enhanced delivery of the radiolabeled drug into the caudate nucleus of rat brain. It was shown that drug concentration was maximal after direct infusion into the brain, up to 10,000 times higher than after intravenous administration. Also, the drug levels were higher after intrathecal and intraventricular infusions in comparison to intravenous delivery. The intrathecal, intraventricular, and convection-enhanced routes produced local distribution patterns with large tissue concentration gradients, which may be relevant to therapeutic applications. It was assumed that brain cells absorb Ara-C, since its loss from brain occurred more slowly than predicted by efflux across the brain-blood barrier. Ara-C was metabolized by the brain with significant accumulation of uracil arabinoside after intravenous administration. Surprisingly, this metabolite was found in the brain after direct infusion as well [93]. Intravenously administered Ara-C showed initial and terminal half-lives of 1.9 and 46.5 min which was in agreement with previously reported values in dogs [94]. Furthermore, the study on dogs and rats demonstrated that the volume of drug distribution is much larger when the drug is administrated into brain white matter in comparison to the infusion of Ara-C into the grey structure [93].

In vitro studies have shown that antitumor activity of tested drugs can be improved when cytotoxic agents are applied in combination. Thus, Vyxenos (CPX-351), the liposomal combination of antineoplastic drugs Ara-C /daunorubicin which is widely used for the treatment of acute myeloid leukemia, was evaluated in many studies before clinical utilization. The development of this formulation was based on the synergistic analyses of Ara-C and daunorubicin in a wide range of leukemia tumor models. In such a way, these two drugs demonstrated retention of synergy on multiple leukemic and solid-tumor cell lines in vitro [95] and proved to be synergistic in murine models of hematological malignancies after intravenous infusion [96]. Additionally, it was shown that the molar ratio of free Ara-C/daunorubicin represented only a small fraction (less than 0.1%) of the encapsulated drug concentrations throughout 40 h due to their leak into the systemic circulation. Also, it was pointed out that uptake of liposomes of Ara-C/daunorubicin decreases as a body weight increases across species [96].

Another preclinical screening of Vyxenos demonstrated notably cytotoxic potential against a wide range of leukemia cell types freshly isolated from patient’s biopsies (peripheral blood or bone marrow) [81]. Carol et al. [97] suggested that Vyxenos may be a promising chemotherapeutic to be utilized in the treatment of acute lymphoblastic leukemia since this formulation has demonstrated significant anti-leukemic activity in vivo against five childhood acute lymphoblastic leukemia xenograft models. Vyxenos treatment of mice provided evidence of significant delay in tumor progression, especially in four B-lineage xenografts in a dose which suggests clinically relevant plasma drug exposure and thus, supported the conduction of an ongoing phase I trial in children with relapsed acute lymphoblastic and acute myeloid leukemia. Also, it is important to note that the pharmacokinetic parameters for Vyxenos are dose-independent in both preclinical and clinical studies [97].

Preclinical testing of efficacy and dependence ratio of Ara-C and daunorubicin in 15 tumor lines in vitro confirmed the superiority of drug delivery system when the molar ratio of Ara-C and daunorubicin is fixed at 5:1. The highest efficacy drug ratio-synergy was achieved in a P388 leukemia model where the drugs were administrated in liposomes at molar ratios ranging from 1:1 to 10:1 for Ara-C and daunorubicin, respectively. The maximal percent of survival of P388 ascites tumor-bearing mice was noted after administration of the liposomal combination at a 5:1 ratio which produced 100% long-term survival. In contrast, only 50% of long-term survival was observed after delivery of the drugs at a 3:1 molar ratio [84]. Tardi et al. [95] clarified that 5:1 molar ratio of Ara-C and daunorubicin in Vyxenos was maintained in the plasma and bone marrow of leukemia-bearing mice for more than 24 h in vivo and thus increased survival of tested animals in comparison to conventional Ara-C and daunorubicin. Also, the molar ratio 5:1 of Ara-C/daunorubicin was maintained in plasma for up to 48 h after infusion to rats and dogs [86].

It was published that Vyxenos, with a mean diameter of 107 nm and strong negative surface potential, consists of gel-phase bilamellar liposomes [98]. Ara-C and daunorubicin interact with Cu(II), and nanoscale liposomes were engineered so that co-encapsulated synergistic molar ratio 5:1 of Ara-C: daunorubicin can form Cu(II) mediated drug loading mechanism. It was confirmed that the interactions of both drugs with Cu(II) gluconate/triethanolamine-based buffer system plays a role in the maintenance of the 5:1 Ara-C: daunorubicin ratio within the Vyxenos. Overall, Cu(II) coordination was found to be critical for the retention of both drugs inside the formulation [86,98].

### 6.2. Clinical Use

Ara-C, a pyrimidine nucleoside-based anticancer drug with arabinose sugar, is in general used for the treatment of hematological malignancies. Inside this wide category, acute myeloid leukemia (AML), acute lymphocytic leukemia [15], non-Hodgkin’s lymphoma (NHL), chronic myelocytic leukemia (blast phase) can be included [99,100]. Ara-C is frequently used in combination with daunorubicin, doxorubicin, or vincristine, and less commonly alone for the treatment of many malignancies [77].

Differently, Ara-C as a liposomal drug is used in lymphomatous meningitis, and this is the only approved indication by Food and Drug Administration (FDA) and EMA (European Medicines Agency) (Figure 2). However liposomal forms of Ara-C are scarcely used if compared to liposomal anthracyclines, which have a pronounced advantage, the reduced cardiotoxicity. This underlines a deficiency in DepoCyt clinical studies for different diseases.

DepoCyt has a long sustained-release due to being encapsulated in multivesicular lipid-based particles. It is directly injectable as a suspension into the cerebrospinal fluid (CSF) via the lumbar sac or via the intraventricular reservoir, slowly over 1–5 min. Depending on the dosing regimen, DepoCyt can be given as scheduled for induction therapy, consolidation therapy, and maintenance. In the first case, the drug is intrathecally-administered every 14 days for 2 doses at weeks 1 and 3. In the second case, DepoCyt is intrathecally-administered every 14 days for 3 doses at weeks 5, 7, 9 followed by one additional dose at week 13. In case of maintenance, patients can be treated intrathecally every 28 days for 4 doses at weeks 17, 21, 25, 29. To diminish side effects or allergic phenomena, DepoCyt is preceded by corticosteroids administration for 5 days before injection [101].

As for all drugs, side effects can also arise with DepoCyt, and serious adverse reaction should be carefully evaluated as early as the first administration. Indeed, the most worrying reaction is arachnoiditis, a burdensome inflammation of the middle layer of membranes of the central nervous system. Arachnoiditis manifests mainly by headache, vomiting, nausea, and fever (it is potentially fatal if untreated) and has been reported as a common adverse event in all studies that tested DepoCyt. Also, neurotoxicity and transient elevations in CSF protein and CSF white blood cells can be observed. From a patient point of view, there are frequent reports of headache, nausea, vomiting, weakness, confusion, pyrexia, fatigue, constipation, back pain, gait abnormal, convulsion and other infrequent symptoms, plus of course arachnoiditis. When concomitant radiation or chemotherapy is associated with DepoCyt in patients with neoplastic meningitis, the risk of adverse effects increases exponentially [101]. Of note that clinicians can limit the severe effects of arachnoiditis with the appropriate administration of drugs such as corticosteroids, antispasmodic drugs, anti-convulsants, and in some cases narcotic pain relievers. Table 2 summarizes the clinical studies of DepoCyt.

These clinical data were obtained from different studies, and as far back as 1993 phase, I studies proposed the human experimentation of DepoCyt in patients with neoplastic meningitis [90,102]. One of these studies reported that single lateral ventricle injection could maintain a therapeutic drug concentration of DepoCyt in the CSF for a period of 9 +/− 2 days, while intra-lumbar administration was maintained for up to 14 days [90]. The authors claimed that DepoCyt permitted patients treatment once every 2 weeks, greatly enhancing drug efficacy (7 patients out of 9 showed cytologic responses). Moreover, the minimum cytotoxic Ara-C concentration (0.1 μg/mL) was observed for a period of >14 days. The same research group produced an additional work that analyzed the clinical effects of DepoCyt: 9 patients were subjected to 1 to 7 cycles in doses ranging from 25 to 125 mg [102]. It has been shown that toxic episodes were transient and reversible (except one) and consequently well tolerated, thanks to malignant cells cleaning in most patients within 3 weeks of initial therapy. Also, DepoCyt did not evade CSF, as measurable plasma concentration of Ara-C or its metabolite uracil arabinoside was virtually absent (detection limit of 0.25 μg/mL). These results gave input to the use of DepoCyt. Indeed they decisively demonstrated a pure pharmacokinetic advantage if compared to unencapsulated intrathecally-administered Ara-C [107]. Later in 1995, the same study group demonstrated that intra-lumbar administration of DepoCyt guaranteed sufficient cytotoxic Ara-C concentrations in both lumbar and ventricular regions and permitted a scheduled drug administration every 14 days [103]. A more wide and randomized study investigated DepoCyt versus free Ara-C in 28 patients with lymphoma and positive CSF cytology [108]. DepoCyt 50 mg every 2 weeks or Ara-C 50 mg twice a week were evaluated for 1 month (induction therapy) and both consolidation (3 months) and maintenance therapy (4 months) were administered to patients without neurologic progression and CSF cytologically negative after treatment. The study demonstrated a response rate of 71% vs. 15%, a time to neurologic progression of 78.5 vs. 42 days and survival trend of 99.5 vs. 63 days, for DepoCyt and free Ara-C, respectively. Also, the better quality of life was sustained by DepoCyt, as measured by the Karnofsky score. Similarly, another randomized trial compared DepoCyt to methotrexate (both administered by intrathecal injection) in 61 patients with neoplastic meningitis [105]. Again, this study underlined as DepoCyt increased the time to neurological progression if compared to methotrexate and emphasized as DepoCyt was administered in less frequent drug-dosing with the same response rate of methotrexate. More recently, the pharmacokinetics of DepoCyt intrathecal administration up to 14 days was evaluated (as part of a phase III study), afresh showing as DepoCyt reached cytotoxic concentrations of Ara-C (>0.02 µg/mL) in CSF with the constant release of Ara-C from the DepoCyt particles [47]. A case report investigating the effects of intrathecally-administered DepoCyt in a diffuse leptomeningeal gliomatosis (due to a glioma-infiltrating leptomeninges) showed that induction and consolidation therapies improved patient clinical status [106]. The result of this case report does not differ substantially from the previous studies, but still highlights once again the effectiveness of DepoCyt in leptomeningeal neoplasia. A retrospective work of Chamberlain (the same author of the first clinical studied on DepoCyt) summarizes the neurotoxicity of DepoCyt in a small group of patients (12.5%) [89]. Bacterial meningitis, chemical meningitis, communicating hydrocephalus, conus medullaris/cauda equina syndrome, decreased visual acuity, encephalopathy, leukoencephalopathy, myelopathy, radiculopathy, and seizures were observed in 120 patients treated for leptomeningeal metastasis. Even if well tolerated, DepoCyt can have serious side effects: this should always prompt stringent clinical observation, waiting for rapid identification of the patients’ subgroup suffering neurological complications. Table 3 summarizes the clinical efficacy of DepoCyt.

There are numerous studies on the therapeutic efficacy of DepoCyt, but in our opinion it is not sufficiently remarked the positive effects of the drug on humans, if compared to other pharmacological options. DepoCyt (encapsulated cytarabine) was superior in multiple aspects compared to free cytarabine. Probably the most important one is related to the clinical practice and to patient benefit. It is not necessary to constantly infuse the drug in CNS if the doctor uses DepoCyt, as the concentration reached is higher and longer enough to guarantee a real advantage [111,112,113]. Undoubtedly, this has decreased infection risk, higher when using a prolonged perfusion of a substance in a such sensitive compartment. Another DepoCyt advantage is that it has been revealed to have a sustained therapeutic concentration in CNS up to 14 days [90,113]. Moreover, in intraventricular dosing, the concentration difference between free and encapsulated cytarabine within 5 h ranged from 1.5 to 116 µg/mL and from 3.8 to 779 µg/mL, respectively. From post-dose day 1 to 14, the concentration ranged from 0.01 to 4.2 µg/mL and from 0.01 to 28.6 µg/mL, respectively. After day 14, the concentration ranged from 0.01 to 8.23 µg/mL and from 0.07 to 213 µg/mL, respectively. Differently in lumbar dosing, the concentration difference between free and encapsulated cytarabine within 5 h ranged from 0.1 to 79 µg/mL and from 2.82 to 1540 µg/mL, respectively. From post-dose day 1 to 14, the concentration ranged from 0 to 0.3 µg/ml and from 0.02 to 5.15 µg/mL, respectively [47]. It is perceivable as DepoCyt can reach a significant concentration in CSF and importantly it maintains this concentration higher than free cytarabine [47]. In vitro studies with multiple cancer cell lines with cytarabine for 24 h showed that the minimum cytotoxic concentration for cytarabine was 0.1 µg/mL, clearly in line with the data above. Another advantage of DepoCyt is half-life elimination: elimination half-life of free cytarabine after intra-CSF injection of DepoCyt is several times longer than after intra-CSF injection of free cytarabine [47]. Taken together, all these data suggest that DepoCyt is firmly better than free cytarabine and advocate for its rational use in patients with neoplastic meningitis.

Of note, in 2017 FDA approved Vyxenos, the dual-drug liposomal combination of daunorubicin and Ara-C (above described) for the treatment of acute myeloid leukemia [114,115,116]. The drug guarantees a synergistic ratio (fixed 5:1 molar ratio of Ara-C and daunorubicin) for over 24 h after intravenous injection in the plasma and is based on CombiPlex platform, i.e., a technology-based system for the development of drug combinations that involves a dual-drug screening. This system is suitable for preclinical evaluation (determining synergistic drug ratios in vitro) of new compounds making the drug development process more rapid and efficient.

As suggested above, liposomal cytarabine was very recently studied in 19 patients affected by neoplastic meningitis to understand if sustained cytotoxic cerebrospinal fluid (CSF) concentrations were still available after 14 days from drug injection [113]. The aim was to compare short peak concentration to lower cytarabine concentration in CSF and the authors effectively showed that the 2 methods are clinically equivalent for liposomal cytarabine.

## 7. Future Perspectives

To date, liposomal Ara-C shows poor usage in human diseases, this may be due to the side effects. However liposomal Ara-C shows a different pharmacological profile compared to Ara-C and reserves great promise for its future. Preclinical data suggest that liposomal Ara-C is ready for subsequent experimentation in the clinic. Only novel clinical trials could lay the basis for greater use of liposomal Ara-C.

## 8. Conclusions

Encapsulated Ara-C is a valid delivery formulation that could represent a great promise as an effective anti-cancer agent. Its current use in lymphomatous meningitis could be potentially expanded. However, liposomal Ara-C has been recently (July 2017) discontinued at least in the USA due to product-specific manufacturing problems. This fact limits the future application of the drug, but not all is lost. New research and impulses dedicated to this agent or class of drugs may reserve a striking impact on cancer therapy.

## Figures and Tables

**Table 1:** Liposomal cytarabine clinical trials (ClinicalTrials.gov).

Phase	Treatment	Disease	Enrollment	Identifier
Phase 2	DepoCyt, methotrexate	Leptomeningeal metastasis of breast cancer	3	NCT00992602
Phase 2	DepoCyt	Lymphomatous or leukemic meningitis	4	NCT00523939
Not Applicable	DepoCyt, sorafenib	Neoplastic meningitis	2	NCT00964743
Phase 3	DepoCyt	Leptomeningeal metastasis of breast cancer	74	NCT01645839
Not Applicable	Vyxenos, liposomal cytarabine, and daunorubicin	Untreated myelodysplastic syndrome, acute myeloid leukemia, acute biphenotypic leukemia, myelodysplastic syndrome	48	NCT01804101
Phase 3	Vyxenos, 7+3 (liposomal cytarabine and daunorubicin)	High risk of acute myeloid leukemia	309	NCT01696084

**Table 2:** Clinical studies of DepoCyt

Study Type	Treatment	Disease	Results	Ref.
Phase I	Intraventricular	Leptomeningeal metastasis	Well-tolerated toxicity, duration of response with a median of over 11 weeks	[102]
Phase I	Intrathecal	Neoplastic meningitis	The therapeutic intra-lumbar concentration of free Ara-C was maintained for up to 14 days	[90]
Phase I	Intra-lumbar	Leptomeningeal metastasis	Extended free Ara-C concentrations	[103]
Phase II	Intrathecal	Leptomeningeal metastasis	Well-tolerated toxicity, systemic high-dose methotrexate + liposomal cytarabine	[104]
Randomized controlled trial	Intrathecal	Neoplastic meningitis	Increased time to neurological progression. Median survival was 105 days with DepoCyt and 78 days with methotrexate	[105]
Open-label study	Intraventricular or lumbar puncture	Leukemia, lymphoma, or solid tumors as part of a phase III study	Extended exposure compared with standard Ara-C	[47]
Case-report	Intrathecal	Secondary diffuse leptomeningeal gliomatosis	Improvement of the clinical status	[106]
Retrospective case series	Intraventricular	Leptomeningeal metastasis	Well tolerate toxicity, in general	[89]

**Table 3:** Clinical efficacy of DepoCyt

Study Type	No. of Patients	Results	Reference
Phase I	9	Duration of response: 2–14 weeks, median 11	[102]
Phase I	12	Therapeutic intralumbar concentration of free cytarabine maintained for 14 days	[90]
Phase I	8	Lumbar and intraventricular max concentration of free cytarabine: 226 and 6.06 mg/L; half-life, 277 and 130 h, respectively	[103]
Phase I	18 children (3–21 years)	Prolonged disease stabilization or response: 14 patients Maximum-tolerated dose: 35 mg	[109]
Randomized controlled trial	31 treated with D, 30 with M	Median survival: 105 days (D), 78 days (M) Median time to neurological progression: 58 (D) vs 30 (M) days Neoplastic meningitis-specific survival: 343 (D) versus 98 (M) days Adverse events: comparable D vs. M	[105]
Open-label study	8	Concentration of free and encapsulated cytarabine in the ventricular and lumbar CSF: 0.01 to 1500 µg/mL	[47]
Case-report	1	Duration of response with D: 6 months	[110]
Retrospective case series	120	D well tolerated, but 12.5% had serious treatment-related neurological complications	[89]
